# A Novel Supraretinacular Endoscopic Carpal Tunnel Release: Surgical Technique, Clinical Efficacy and Safety (A Series of 48 Consecutive Cases)

**DOI:** 10.1016/j.jhsg.2021.06.011

**Published:** 2021-08-13

**Authors:** Kok Kheng Teh, Jayaletchumi Gunasagaran, Ch’ng Hwei Choo, Tunku Sara Ahmad

**Affiliations:** ∗ Department of Orthopaedic Surgery, Faculty of Medicine, National Orthopaedic Centre of Excellence for Research & Learning, University of Malaya, Kuala Lumpur, Malaysia; † Sunway Medical Centre, Selangor, Malaysia; ‡ Sunway University, Selangor, Malaysia

**Keywords:** Carpal tunnel surgery, Carpal tunnel syndrome, Endoscopic carpal tunnel release, Endoscopic surgery, Supraretinacular endoscopic carpal tunnel release

## Abstract

**Purpose:**

Endoscopic carpal tunnel release has been shown to have a shorter recovery period than open surgery. This study was aimed at assessing the efficacy and possible clinical complications of a novel supraretinacular endoscopic carpal tunnel release technique.

**Methods:**

A total of 50 cases involving 46 patients were evaluated in this prospective study, in which all surgeries were performed by a single surgeon between 2016 and 2018. The patients were evaluated preoperatively; at 3, 7, and 14 days after surgery; and at 1, 3, and 6 months after surgery. The effectiveness of the surgery was evaluated using pinch and grip strengths, modified table test, visual analog scale pain score, the Disabilities of the Arm, Shoulder and Hand, the Boston Carpal Tunnel Questionnaire symptom severity scale, and the Boston Carpal Tunnel Questionnaire functional status scale. The Friedman test and Wilcoxon signed-rank test were used for a statistical analysis.

**Results:**

At 6 months after the surgery, all measured parameters showed improvements. The pinch strength score improved from 2.29 kg before the surgery to 2.96 kg 6 months after the surgery (*P* = .003), the grip strength score improved from 12.10 kg to 13.98 kg (*P* = .028), the modified table test score increased from 6.55 kg to 8.76 kg (*P* < .001), the visual analog scale score decreased from 6.31 to 0.52 (*P* < .001), the Disabilities of the Arm, Shoulder and Hand score reduced from 41.66 to 14.10 (*P* < .001), and the Boston Carpal Tunnel Questionnaire symptom severity scale and the Boston Carpal Tunnel Questionnaire functional status scale scores reduced from 2.68 to 1.51 (*P* < .001) and from 2.56 to 1.44 (P < .001), respectively. There were no serious injuries or complications reported in this series.

**Conclusions:**

This new supraretinacular endoscopic carpal tunnel release technique was shown to be efficacious in this series.

**Type of study/level of evidence:**

Therapeutic IV.

Both open carpal tunnel release and endoscopic carpal tunnel release (ECTR) are highly effective treatments for carpal tunnel syndrome (CTS). Nevertheless, ECTR has an advantage of the early recovery of hand function, which allows a shorter time of return to work.^1^^,^^2^

Since Okutsu’s first description of ECTR in 1987, many endoscopic techniques have been developed, but most of the ECTR literature pertains to the techniques described by Agee et al^3^ and Chow.^1^^,^^4^ Both these techniques employed the carpal tunnel as the portal for carpal tunnel release. However, these techniques are associated with a higher risk of nerve injury.^2^

In contrast, Ip et al^5^ and Ecker et al^6^ introduced the use of a supraretinacular approach to release the transverse carpal ligament. This technique used the instrumentation that was designed for endoscopic cubital tunnel release.

Previously, we designed a smaller instrumentation and a technique to release the transverse carpal ligament through a similar approach.^7^ In that study, we described a new technique using the supraretinacular approach and found that by abiding to a safe zone, we could perform ECTR without causing any injuries to vital structures in the wrist—namely, the median nerve, superficial palmar arch, and flexor tendons—or violation of the Guyon canal.

In this follow-up clinical study, we aimed to evaluate whether this new technique would be an efficacious treatment for CTS and to identify potential clinical and technical complications. Our study hypothesis was that the supraretinacular approach would be a safe and effective means to release the carpal tunnel.

## Materials and Methods

This series comprised 50 consecutive wrists of 46 patients who had been diagnosed with CTS by a specialist based on a standard clinical history and physical examination with or without a nerve conduction study in a tertiary university hospital from January 2016 to December 2018. Four patients had bilateral CTS. Ethics approval was obtained from the medical ethics committee of the university to conduct this study (ethics committee reference number: 1182.36).

The demographic data of the patients are summarized in Table 1. All 50 wrists were operated upon by a single surgeon. Two patients (2 wrists) were lost to follow-up. Thus, finally, 48 wrists of 44 patients were included in this study.

**Table:** Demographic Data

Characteristics	n (%) or Mean ± SD
Age, y	59 + 14
Sex	
Male	9 (20.5)
Female	35 (79.5)
Wrist involved	
Right	19 (39.6)
Left	29 (60.4)
Patients with wrist involved	
Single	40 (90.9)
Both	4 (0.09)

All the cases were evaluated using the following validated outcome instruments and quantitative measurements: pinch strength, grip strength, modified table test value, visual analog scale (VAS) score, Disabilities of the Arm, Shoulder and Hand (DASH) score, Boston Carpal Tunnel Questionnaire symptom severity scale (BCTQ-S) score, and Boston Carpal Tunnel Questionnaire functional status scale (BCTQ-F) score. The cases were evaluated before the surgery; 3, 7, and 14 days after the surgery; and 1 month, 3 months, and 6 months after the surgery.

Previously, we modified the original table test to assess the intensity of pillar pain after surgery.^8^ The patients were asked to place their operated hand on a weighing scale, leaning their weight on their palm and pushing down as hard as they could. The maximum weight was recorded during every follow-up.

### Endoscope device

**Figure 1:**
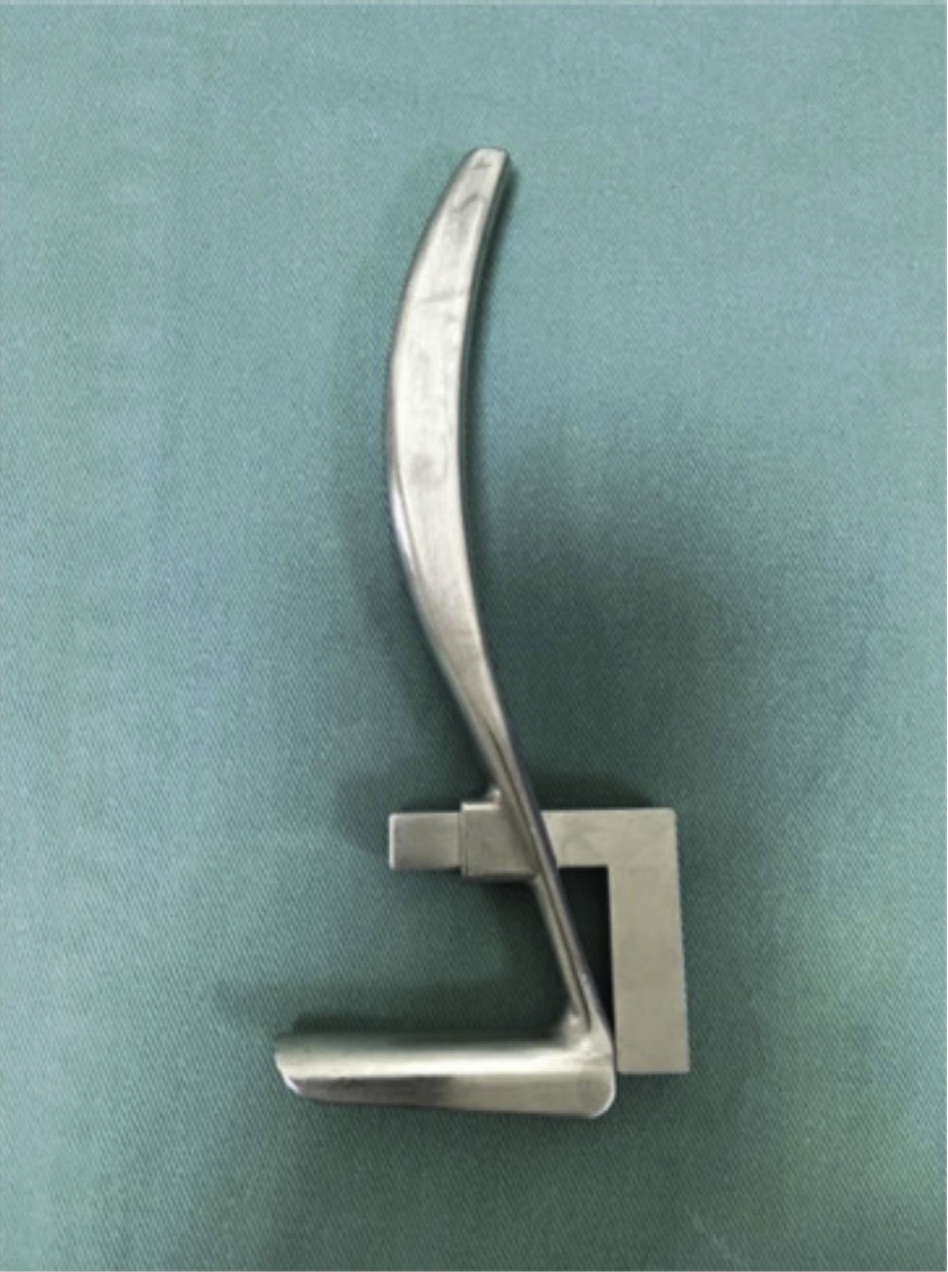
A supraretinacular retractor with L-bar for endoscope placement.

The newly designed supraretinacular retractor (Fig. 1) was used along with either a 2.4-mm or a 2.7-mm endoscope. The endoscope was held in place using an L-bar, which enabled it to be perched at the top of the supraretinacular retractor’s dome. This device was designed by the main author and produced by a contract instrument manufacturer fully funded by the main author.

### Surgical technique

**Figure 2:**
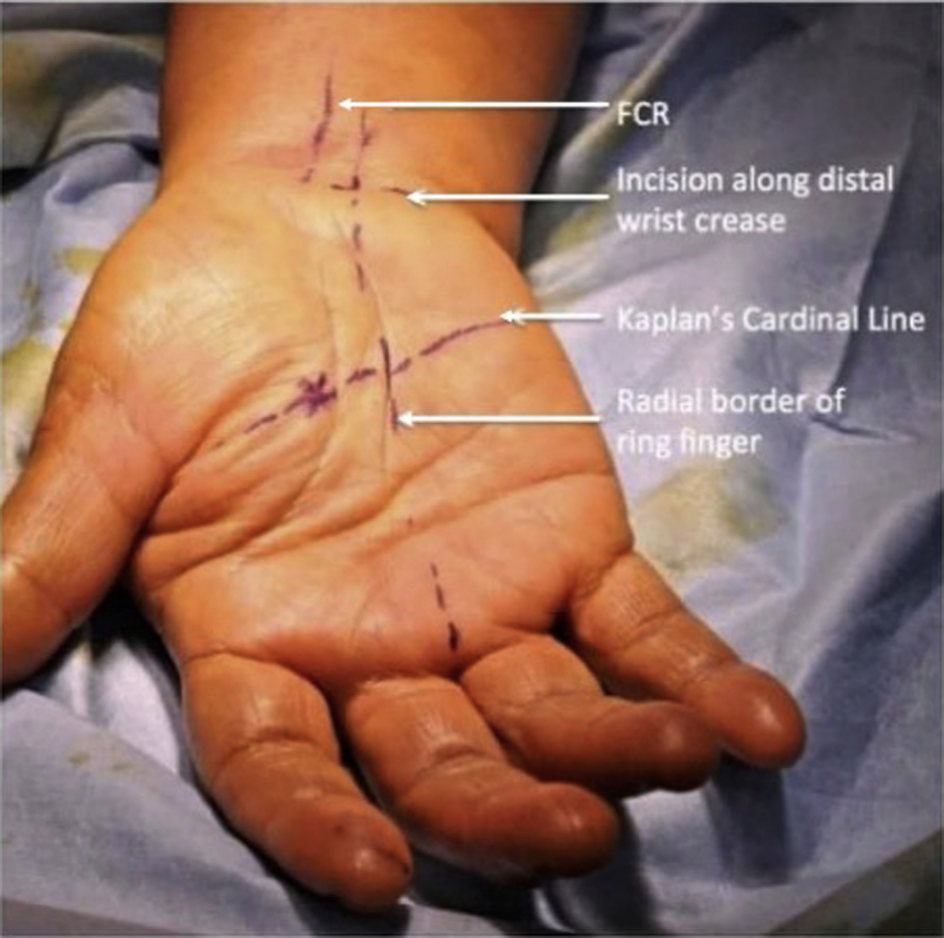
Surface anatomy markings for safe zone. FCR, flexor carpi radialis.

**Figure 3:**
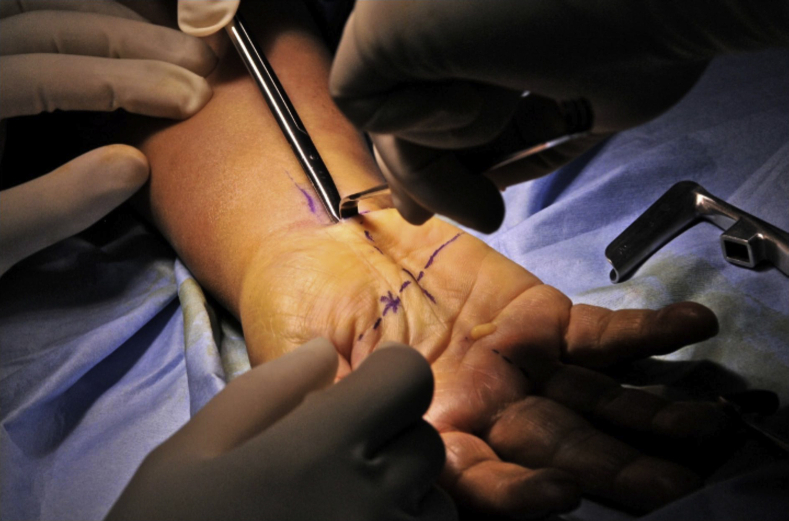
A supraretinacular plane was developed between palmar and the transverse carpal ligament via a blunt dissection using scissors.

**Figure 4:**
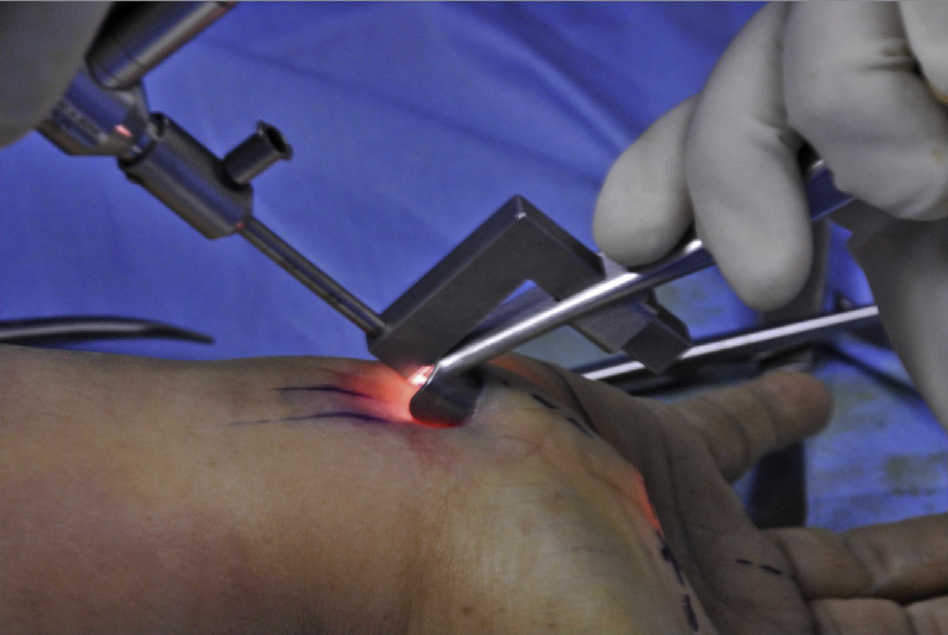
The endoscope was inserted using the L-bar to visualize the supraretinacular plane.

**Figure 5:**
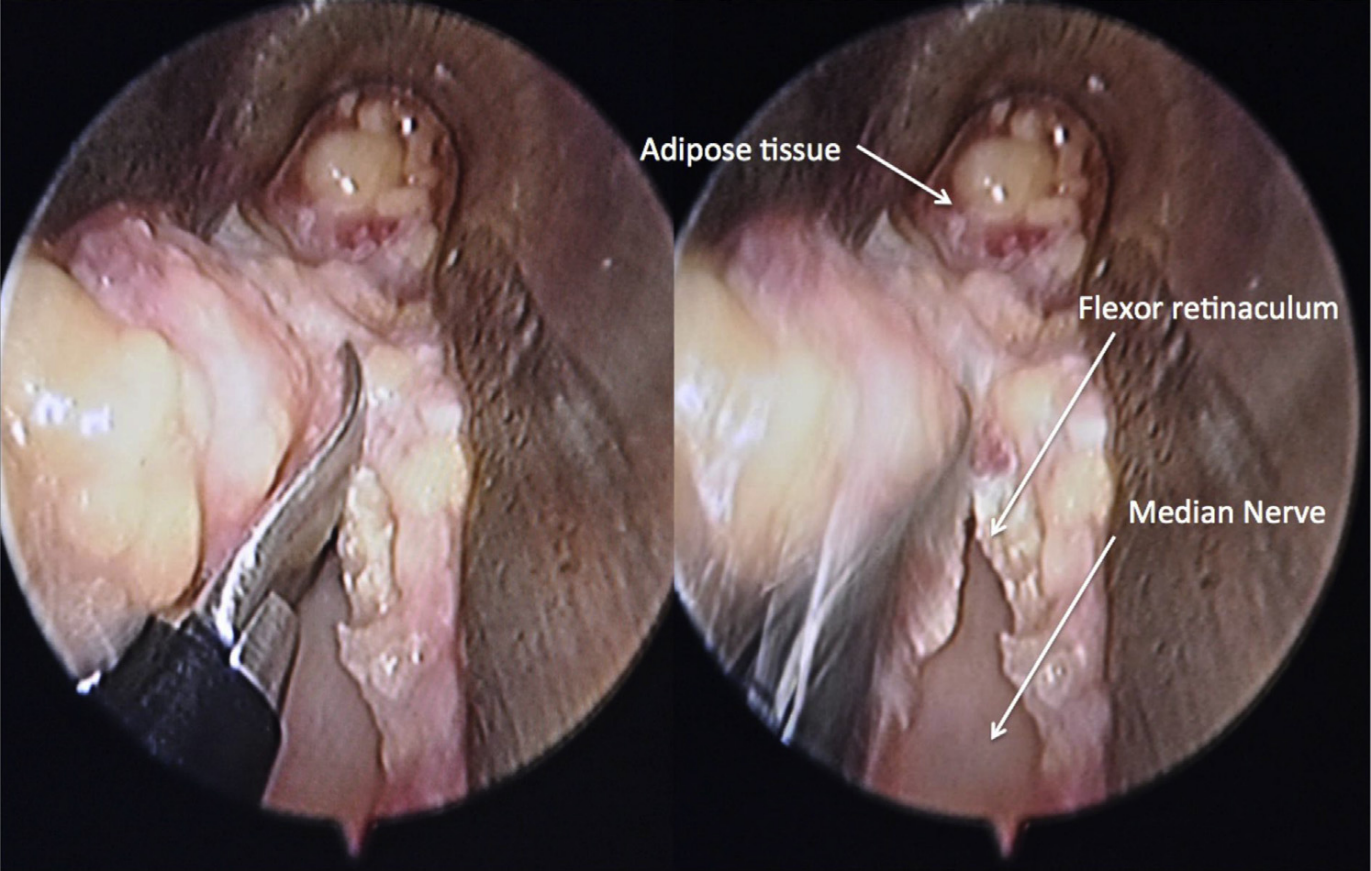
Flexor retinaculum was visualized at the floor and released distally until the fat pad was reached.

**Figure 6:**
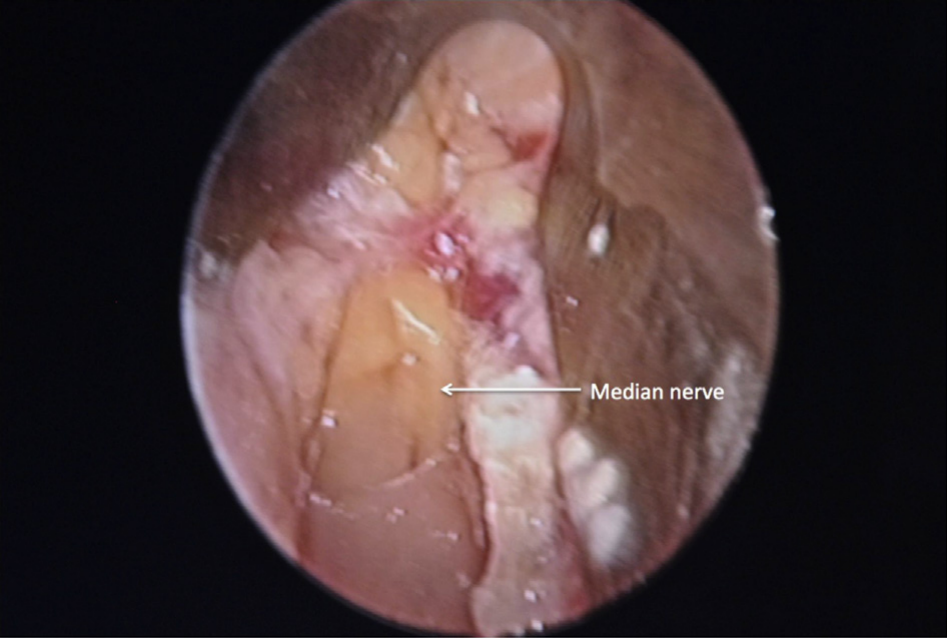
After the release of the flexor retinaculum, the median nerve with the overlying fat pad was used to signify complete release.

All the cases in the series were operated upon under local anesthesia, with a pneumatic tourniquet on the upper arm of the operated side, except for the first case. The first case was operated upon under general anesthesia as a safety measure. Surface markings for the flexor carpi radialis, palmaris longus, Kaplan cardinal line, and radial border of the ring finger were made before the surgery (Fig. 2). The surgery was performed with a 2-cm transverse incision on the radiocarpal wrist flexion crease between the palmaris longus tendon and the ring finger’s ulnar border. Tenotomy scissors were used to create a supraretinacular space that cut along the axial line between the middle and ring fingers and ended at the Kaplan cardinal line (Fig. 3). A 2–3-cm-wide tunnel was made to allow the supraretinacular retractor to be inserted above the retinaculum. The L-bar was used to hold the endoscope, which allowed the visualization of the transverse carpal ligament (Fig. 4). A dissection of the proximal part of the transverse carpal ligament was then performed using tenotomy scissors to visualize the median nerve underneath. The transverse carpal ligament was released via a series of small sequential cuts using tenotomy or laparoscopic scissors till the ligament’s distal edge (Fig. 5). Completion of the division of the transverse carpal ligament was confirmed by visualizing the overlying fat pad that covers the median nerve (Fig. 6). The antebrachial fascia was released with a proximal dissection under direct vision. The portal site was sutured and covered with a simple dressing and bandaged. The patients were then followed up on days 3, 7, and 14 and after 1, 3, and 6 months. They were assessed clinically for any surgical complications, such as wound infection, dehiscence, neurovascular injury, or worsening of symptoms. They were also assessed objectively using the grip strength measurements, modified table test, and functional scores of the VAS, DASH, and Boston Carpal Tunnel Questionnaire.

### Statistical methods

All data were analyzed using SPSS, version 26.0 (IBM Corp). The demographic data were analyzed with descriptive statistics. Nonparametric tests were chosen for the analysis of outcome measures because the samples were not normally distributed. The Friedman test was used for multiple comparisons of paired data to assess differences between preoperative and various postoperative time points for each outcome measured. Subsequently, the Wilcoxon signed-rank test was used to compare the distribution of each outcome across the 2 time points (preoperative and postoperative). A 2-tailed *P* value of <.05 was considered significant.

## Results

The mean age of the patients was 59 (SD 14, range 30–87) years. There were 35 women and 9 men. Nineteen patients had right wrist involvement, and 29 had left CTS (Table 1).A total of 48 cases came back for follow-up, with 2 cases lost to follow-up, on days 3, 7, and 14 and at 1, 3, and 6 months. There were no neurovascular or tendon injuries or other major complications. However, 1 patient had excessive bruising over the arm because of tourniquet-induced pressure, which resolved without any intervention.

**Figure 7:**
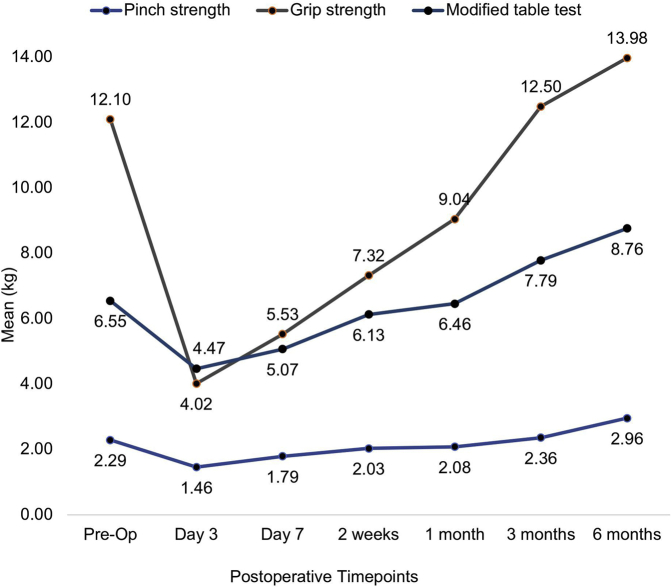
Mean values of grip strength, pinch strength, and modified table test (kg) during each visit.

The parameters of the measured outcomes were as follows:

The pinch strength value was 2.29 kg (SD, 1.62) before the surgery, reduced to 1.46 kg (SD, 1.04) on day 3 but improved over time to 2.96 kg (SD, 2.25) at 6 months after the surgery (*P* = .003) (Fig. 7).

The grip strength value was 12.10 kg (SD, 8.36) before the surgery, reduced to 4.02 kg (SD, 4.85) on day 3 but improved over time to 13.98 kg (SD, 7.58) at 6 months after the surgery (*P* = .028) (Fig. 7).

The modified table test was used to measure pillar pain before and after the surgery. The mean modified table test value was 6.55 kg (SD, 3.89) before the surgery, reduced to 4.47 kg (SD, 3.28) on day 3 but gradually increased to 8.76 kg (SD, 5.28) at 6 months after the surgery (*P* < .001) (Fig. 7).

**Figure 8:**
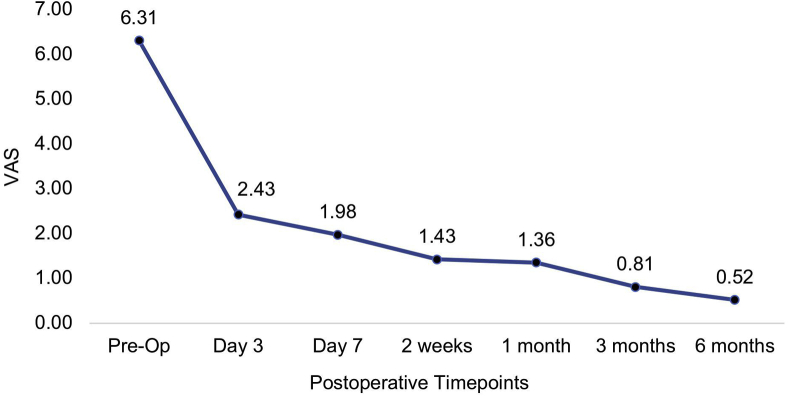
Mean values of the VAS during each visit. Pre-Op, preoperative.

The VAS score decreased from 6.31 (SD, 1.97) before the surgery to 2.43 (SD, 2.47) on day 3 and continued to improve to a value of 0.52 (SD, 1.61) at 6 months after the surgery (*P* < .001) (Fig. 8).

**Figure 9:**
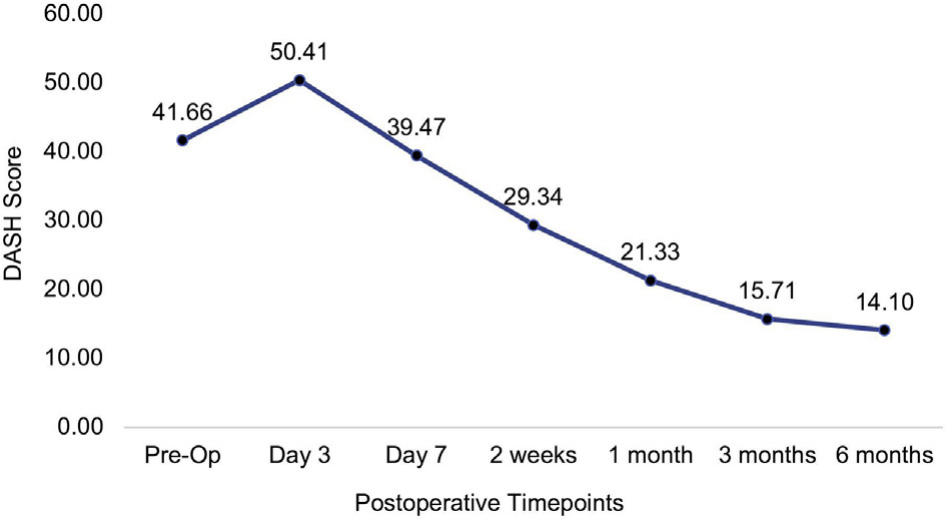
Mean values of the DASH during each visit.

The DASH score was 41.66 (SD, 19.90) before the surgery, increased to 50.41 (SD, 22.20) initially on day 3 but dropped below the preoperative score to 39.47 (SD, 20.89) on day 7 and continued to drop further to 14.10 (SD, 21.28) at 6 months after the surgery (*P* < .001) (Fig. 9).

**Figure 10:**
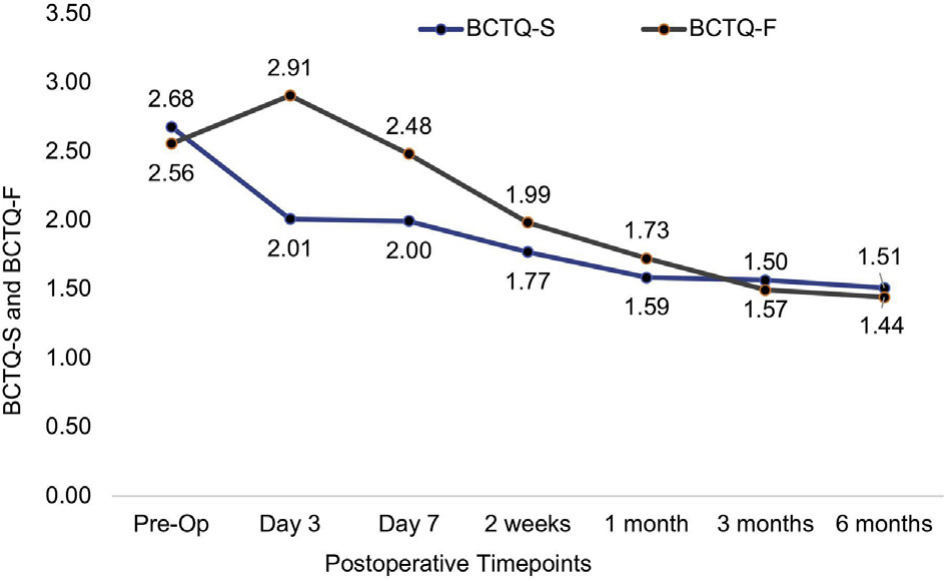
Mean values of the BCTQ-S and BCTQ-F during each visit.

Both the BCTQ-S and BCTQ-F scores improved from 2.68 (SD, 0.74) before the surgery to 1.51 (SD, 0.86) at 6 months after the surgery and from 2.56 (SD, 0.92) before the surgery to 1.44 (SD, 0.83) at 6 months after the surgery, respectively, (*P* <.001) (Fig. 10).

## Discussion

In summary, the patients in this series had symptomatic improvement as early as 3 days after the surgery, as evidenced by the improvement of the VAS and BCTQ-S scores beyond the preoperative values. Furthermore, the BCTQ-F and DASH scores improved beyond the preoperative values by 7 days after the surgery. The measured pinch strength, grip strength, and modified table test values showed an improvement beyond the preoperative values by 3 months after the surgery.

Endoscopic carpal tunnel release was introduced as a new surgical treatment for CTS in the late 1980s. To date, the 2 most common ECTR techniques are the dual-portal technique described by Chow^4^ and the single-portal technique described by Agee et al.^3^ Comparative clinical studies have shown that ECTR resulted in less postoperative pain, quicker recovery of grip and pinch strengths, and earlier return to work than open carpal tunnel release.^2^^,^^9^, ^10^, ^11^

However, these techniques may be associated with higher risk of injuring the median nerve with the infraretinacular or transcarpal tunnel approach.^2^ Endoscopic carpal tunnel release is associated with injuries of the superficial palmar arch, ulnar neurovascular bundle, median nerve, and common digital nerve.^12^ One of the main reasons causing these complications is the anatomy of the carpal tunnel itself. The carpal tunnel height at the proximal wrist where the wrist portal is made for most ECTR techniques is about 12 mm, while that at the distal end it is about 10 mm.^13^ Therefore, inserting a 6-mm-diameter instrument (4-mm endoscope plus 2-mm sleeve) into a tight and diseased carpal tunnel may increase the risk of injuring its content.

**Figure 11:**
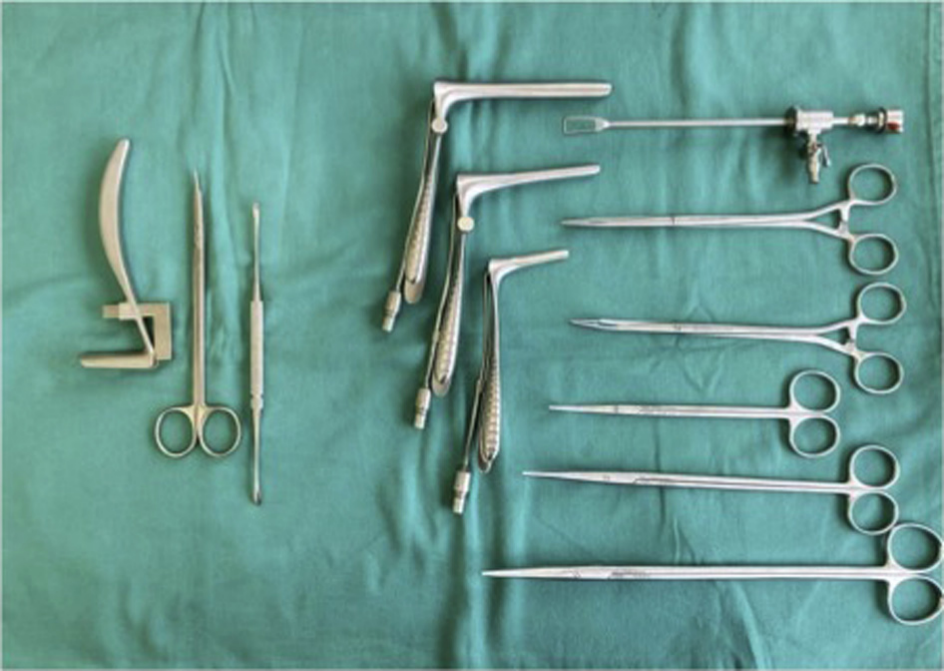
Side-by-side comparison of the SRECTR surgical equipment set and Hoffman endoscopic cubital tunnel release set. SRECTR, supraretinacular endoscopic carpal tunnel.

In contrast to the above techniques, Ip et al^5^ and Ecker et al^6^ presented a supraretinacular endoscopic carpal tunnel technique. In this technique, the endoscope and instrumentations are inserted superficial to the flexor retinaculum. This technique allows the visualization of the transverse carpal ligament without putting the median nerve at higher risk during the surgery.^14^ This alternative approach allows better visualization of the median nerve and muscles over the flexor retinaculum’s palmar surface as well. Both the techniques used instruments that were initially designed for endoscopic cubital tunnel release. The instruments are long and use a 4.0-mm endoscope with large Mayo scissors. Our technique employs a similar supraretinacular approach using a smaller instrumentation. We believe that this may be beneficial to the patient by reducing the possibility of collateral damage to surrounding tissues and causing less scarring after surgery. Ip et al^5^ reported 4 cases of pillar pain and 2 cases of hypertrophic scar in their series of 10 patients. In our series, there were no cases of hypertrophic scar or pillar pain. The modified table test value, used to assess pillar pain, improved beyond the preoperative value at 3 months after the surgery and continued to improve at 6 months after the surgery. Our series provides evidence that this new technique, using less and a smaller instrumentation, is feasible and efficacious in carpal tunnel release. Figure 11 shows the 2 different instrumentations side by side for size comparison, wherein on the left are the supraretinacular endoscopic carpal tunnel instruments and on the right is the Hoffman cubital tunnel release set that was used by Ip et al^5^ and Ecker et al^6^ for their ECTRs.

This series of 48 cases showed that this new technique is an efficacious method for the treatment of CTS. The VAS and BCTQ-S scores showed a significant improvement as early as 3 days after the surgery. Both the DASH and BCTQ-F scores showed an improvement by day 7 from the preoperative scores, although not statistically significant, but achieved statistical significance at the second week of follow-up. The pinch strength, grip strength and modified table test values showed an improvement beyond the preoperative values at 3 months, although only the modified table test value achieved statistical significance. There were no serious surgical complications in terms of nerve, vessel, or tendon injuries in this series. Only 1 patient had bruising secondary to tourniquet-induced pressure on the arm in this series, which resolved without any intervention. Since that case, most patients have undergone surgery under local anesthesia with adrenaline or epinephrine. Most cases were performed with tourniquet inflation as a surgeon preference, but for patients who could not tolerate a tourniquet, the surgery was completed without it (wide-awake local anesthetic no tourniquet).

This study provides evidence that this new supraretinacular endoscopic carpal tunnel technique is an efficacious treatment for CTS and may reduce the risk of transient nerve injury compared with transcarpal tunnel endoscopic techniques. There were no major complications associated with the technique.

The limitation of this study is that it is a single-surgeon series. The study was designed to be a single-surgeon series to ensure consistency of the new technique for the safety of the trial subjects. The instrumentations were designed and the technique developed by the primary author. We believe that any surgeon who has some experience in endoscopic surgery and open carpal tunnel release would be able to perform this surgery comfortably within their first 5 cases. Further randomized controlled trials should be conducted to compare this technique with the existing techniques in terms of effectiveness and safety.
